# Post-translational modifications of Keap1: the state of the art

**DOI:** 10.3389/fcell.2023.1332049

**Published:** 2024-01-08

**Authors:** Yunjia Song, Ying Qu, Caiyun Mao, Rong Zhang, Deyou Jiang, Xutao Sun

**Affiliations:** ^1^ Department of Pharmacology, School of Basic Medical Sciences, Heilongjiang University of Chinese Medicine, Harbin, China; ^2^ Department of Typhoid, School of Basic Medical Sciences, Heilongjiang University of Chinese Medicine, Harbin, China; ^3^ Department of Synopsis of the Golden Chamber, School of Basic Medical Sciences, Heilongjiang University of Chinese Medicine, Harbin, China

**Keywords:** Keap1, Nrf2, post-translational modification, oxidative stress, biomarker

## Abstract

The Keap1-Nrf2 signaling pathway plays a crucial role in cellular defense against oxidative stress-induced damage. Its activation entails the expression and transcriptional regulation of several proteins involved in detoxification and antioxidation processes within the organism. Keap1, serving as a pivotal transcriptional regulator within this pathway, exerts control over the activity of Nrf2. Various post-translational modifications (PTMs) of Keap1, such as alkylation, glycosylation, glutathiylation, S-sulfhydration, and other modifications, impact the binding affinity between Keap1 and Nrf2. Consequently, this leads to the accumulation of Nrf2 and its translocation to the nucleus, and subsequent activation of downstream antioxidant genes. Given the association between the Keap1-Nrf2 signaling pathway and various diseases such as cancer, neurodegenerative disorders, and diabetes, comprehending the post-translational modification of Keap1 not only deepens our understanding of Nrf2 signaling regulation but also contributes to the identification of novel drug targets and biomarkers. Consequently, this knowledge holds immense importance in the prevention and treatment of diseases induced by oxidative stress.

## Introduction

The concept of “oxidative stress” refers to the state of imbalance between oxidants and antioxidants, as well as the impairment of repair mechanisms, resulting in the disruption of redox signaling, and potentially leading to molecular damage. It is crucial to differentiate between eustress (beneficial stress) and distress (harmful stress) in this context. Oxidative eustress denotes physiological deviations from the optimal redox equilibrium, often referred to as the “ideal state of wellbeing”. However, when oxidative challenge surpasses certain thresholds, it transitions into oxidative distress, which is associated with the occurrence of biomolecular harm (Murphy et al., 2022; Sies et al., 2022).

Reactive oxygen species (ROS) is a term encompassing a diverse range of reactive oxygen deviates, including free radicals such as superoxide anion radical (O_2_^–^), hydroxyl radical (HO), peroxyl radical (ROO) and alkoxyl radical (RO), as well as non-radical molecules like hydrogen peroxide (H_2_O_2_), ozone (O_3_), organic hydroperoxides (ROOH) and peroxynitrite (ONOO^−^). Among these, H_2_O_2_ and O_2_^–^ are recognized as the primary ROS involved in the regulation of biological activities through redox mechanisms. The steady-state physiological flux of H_2_O_2_ to specific protein cysteine residues, leading to the formation of sulfenic acid, results in reversible oxidation and subsequently alters protein activity, localization, and interactions. This process plays a crucial role in coordinating various cellular and organ processes, including cell proliferation, differentiation, migration, and angiogenesis. The mitochondrial electron transport chain (ETC.) generates O_2_^–^, which is subsequently converted to H_2_O_2_ and other oxidants. The presence of O_2_^–^ can disrupt the iron-sulfur (Fe-S) cluster in the aconitase, an enzyme of citric acid cycle, thus affecting cell mitochondrial activity. Additionally, O_2_^–^ can activate uncoupling protein 1 (UCP1), providing protection against excessive membrane potential. Furthermore, O_2_^–^ can cause inactivation of the Fe-S form of mitochondrial GRX2 (Sies and Jones, 2020; Murphy et al., 2022; Sies et al., 2022).

The Keap1 protein serves as a crucial regulator with significant biological implications within the cell (Song et al., 2020). In its capacity as an intracellular regulator, the Keap1 protein plays a pivotal role in modulating oxidative stress. The examination of the structure and function of the Keap1 protein suggests that it modulates oxidative stress through its interactions with other proteins. Additionally, it primarily regulates the expression of antioxidant response genes by controlling the stability of the transcription factor Nrf2 (McMahon et al., 2003; Kim et al., 2014; Baird and Yamamoto, 2020; Kopacz et al., 2020). Specifically, it achieves this by employing post-translational modifications (PTMs) to regulate the signaling associated with redox (Katsuragi et al., 2016; Bellezza et al., 2018; Wei et al., 2019; Yu and Xiao, 2021). PTM serves as a crucial intracellular mechanism for the regulation of protein function, encompassing alkylation, glycosylation, glutathionylation, S-sulfhydration, and other modifications. This mechanism plays a pivotal role in maintaining the stability and functionality of the Keap1 protein, thereby governing the oxidative stress response (Zhang et al., 2005; Bollong et al., 2018; Liu et al., 2022).

Furthermore, it is now feasible to develop a variety of compounds that interfere with the inhibitory effect of Keap1 on Nrf2 to combat oxidative stress-related diseases (Filomeni et al., 2015; Mou et al., 2020). Therefore, a comprehensive exploration of the PTMs of the Keap1 protein and its involvement in oxidative stress will yield fresh insights into comprehending the molecular mechanisms of oxidative stress, while also offering novel targets and strategies for the prevention and treatment of associated diseases. In this review, we overview the molecular structure, function, PTMs and regulatory mechanisms of Keap1.

## Structure and function of Keap1 protein

### Structure of Keap1 protein

The Keap1 protein is a polypeptide chain consisting of about 625 amino acids, with a molecular mass of 70 kDa, primarily located in the cytoplasm (Ulasov et al., 2022). Its structural composition encompasses five distinct domains, of which the BTB/POZ and Kelch domains are key conserved domains (Cai et al., 2021). The presence of these conserved domains confers specific functions and regulatory capabilities to the Keap1 protein (Koonin et al., 1992). Notably, the BTB/POZ domain exhibits the ability to interact with various proteins and modulate oxidative stress. Additionally, it plays a role in maintaining the stability of Nrf2 by binding to other proteins, thereby exerting control over the expression of genes associated with the antioxidant response (Zipper and Mulcahy, 2002). Furthermore, it plays a crucial role in mediating the interaction between the Keap1 protein and other regulatory factors, thereby exerting further influence on oxidative stress regulation (Itoh et al., 1997; Cullinan and Diehl, 2004). The Kelch domain, which consists of multiple repetitive units, adopts a β-helix conformation with a distinctive structure, and serves as a vital domain within Keap1 (Zipper and Mulcahy, 2002). The Kelch domain possesses the ability to interact with Nrf2, thereby governing both the degradation of Nrf2 and the expression of antioxidant response genes associated with it. Particular amino acid residues within the Kelch domain play a crucial role in the binding of Keap1 protein to Nrf2 and can be modulated by PTMs (Yamamoto et al., 2018).

In summary, the Keap1 protein exhibits a complex structure comprising various conserved domains, notably the BTB/POZ domain and Kelch domain, which govern the modulation of oxidative stress via interactions with other proteins. Consequently, comprehending and investigating the functionality of the Keap1 protein and its involvement in oxidative stress is of paramount importance.

### Function of Keap1 protein

As a crucial regulator, the Keap1 protein assumes a pivotal role in intracellular oxidative stress, with its interaction with the Nrf2 protein serving as a significant mechanism in the context of oxidative stress (Keum and Choi, 2014; Lu et al., 2016). The main function of Keap1 is to regulate the expression of a series of antioxidant response genes by regulating the stability of the transcription factor Nrf2, and these genes codes for proteins capable of scavenging intracellular free radicals and protect cells from oxidative damage (Itoh et al., 1999; Dhakshinamoorthy and Jaiswal, 2001; Furukawa and Xiong, 2005). More specifically, Keap1 drives the Nrf2 protein to ubiquitination and then degradation via binding to the Neh2 domain of Nrf2 (Pandey et al., 2017). When Nrf2 escapes the degradation of Keap1, it can enter the nucleus and bind to the promoter regions of antioxidant response genes, thereby regulating the genes expression (Zipper and Mulcahy, 2002; Suzuki et al., 2019). Moreover, the role of the Keap1 protein is intricately linked to its structural composition, encompassing five distinct domains, several of which are essential conserved domains, including BTB/POZ and Kelch. These domains significantly contribute to the functionality of the Keap1 protein. Specifically, the Kelch domain facilitates interaction with the Nrf2 protein, thereby regulating its stability within the cytoplasm (Lo and Hannink, 2006).

Nrf2, a pivotal transcription factor, possesses the ability to bind to antioxidant response elements (AREs) to govern the transcriptional activation of gene promoters associated with numerous downstream antioxidant enzymes and phase II detoxification enzymes, and then plays the function of regulating redox balance, drug metabolism and excretion, energy metabolism, iron metabolism, amino acid metabolism, cell autophagy, proteasome degradation, DNA repair and mitochondrial physiology (Tonelli et al., 2018). When cells are exposed to oxidative stress, the Keap1 protein undergoes PTMs such as S-sulfhydration and alkylation. These PTMs disrupt the interaction between Nrf2 and Keap1, allowing Nrf2 to translocate into the nucleus and bind to the promoter region of antioxidant responsive genes (de Freitas Silva et al., 2018; Tu et al., 2019; Ulasov et al., 2022). The resulting antioxidant response proteins, which include antioxidant enzymes like superoxide dismutase, glutathione reductase, and catalase (Sajadimajd and Khazaei, 2018; Yuan et al., 2021), as well as phase II detoxifying enzymes like glutathione S-transferase (GST), NADPH quinone oxidoreductase, gamma-glutamylcysteine synthetase (GGS) and heme oxygenase-1 (HO-1), and translation detoxification enzymes like glutathione synthetase (GSS) (Alam et al., 1999; Kobayashi and Yamamoto, 2005), possess the capability to safeguard cells against oxidative damage by scavenging intracellular free radicals and maintaining intracellular redox balance.

In addition to its role in regulating antioxidant response genes, the Keap1 protein exerts influence on oxidative stress through its interactions with other pertinent proteins. Notably, the Keap1 protein has been observed to interact with the p62 protein, thereby promoting the initiation of the autophagic process. This autophagy pathway serves to selectively eliminate misfolded or impaired intracellular proteins and organelles, complementing the Nrf2-dependent modulation of antioxidative responses. Consequently, this interplay facilitates supplementary defense mechanisms against cellular oxidative stress (Moscat and Diaz-Meco, 2009; Itoh et al., 2015). When the production of ROS surpasses the cellular antioxidant capacity, cells must eliminate dysfunctional mitochondria that contribute to excessive ROS production. The Kelch domain of Keap1 has the ability to interact with mitochondrial proteins, thereby regulating the generation of mitochondrial superoxide and subsequently modulating the cellular response to oxidative stress (Itoh et al., 1999; Zeb et al., 2021). Additionally, the interaction between Keap1 and Akt protein can regulate the activity of the Akt signaling pathway, thereby influencing cell survival and apoptosis (Yuan et al., 2021).

Together, these results indicate that Keap1 protein plays a significant role in cellular regulation. Further investigation of the function of Keap1 protein will help to reveal the molecular mechanism of oxidative stress and provide new ideas and strategies for the treatment and prevention of related diseases.

## Post-translational modifications of Keap1 protein

### Ubiquitination

Among the post-translational modifications (PTMs) of Keap1 protein, ubiquitination is an indispensable process. Ubiquitination, a common protein modification method, can regulate the function, stability and localization of target proteins by way of covalently binding to ubiquitin (Ub) proteins, and link to many biological processes such as apoptosis and inflammation (Kirkin and Dikic, 2007; Liu et al., 2021a). Ubiquitination of Keap1 is regulated by E1 ubiquitin-activating enzymes (E1 enzyme), E2 ubiquitin-conjugating enzymes (E2 enzyme) and E3 ubiquitin ligase (E3 enzyme), the process is as follows: firstly, the E1 enzyme combines ATP to generate the ubiquitin-AMP intermediate; then, the E2 enzyme forms the ubiquitin-protein covalent binding by linking ubiquitin-AMP intermediates to cysteine residues on the target protein; finally, the E3 enzyme completes the process of ubiquitination by recognizing the target protein and catalyzing the covalent binding of ubiquitin (Park et al., 2020).

**FIGURE 1:**
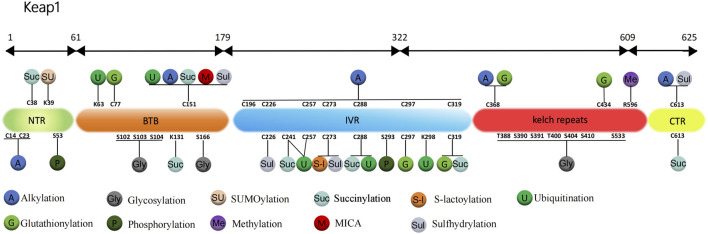
Structure and post-translational modification sites of the human Keap1 protein. NTR: N-terminal region; Intervention zone IVR: intervening region; BTB: Broad complex, Tramtrack and Bric-à-Brac; CTR: C-terminal structural domain; K, Lys: lysine; S, Ser: serine; C, Cys: cysteine; T, Thr: threonine; R, Arg: arginine.

For the Keap1 protein, its ubiquitination mainly occurs at some specific positions. Mutants of the Keap1 protein, 125 to 127 and 162 to 164 sites mutated to alanine, showed increased levels of ubiquitination. And these mutant Keap1 proteins were unable to cooperate with Cul3 for ubiquitination of the Nrf2 protein and repression of steady-state levels of Nrf2. In addition, the mutant Keap1 proteins were impaired in their ability to downregulate Nrf2-dependent gene expression (Zhang et al., 2004). Zhang et al. (2005) further found that the initial event for inhibition of Keap1 by both quinone and sulforaphane-induced oxidative stress is a chemical modification on Cys151, which decreases efficient assembly of Nrf2-bound Keap1 into a Cul3-dependent ubiquitin ligase complex. However, prolonged oxidative stress, may result in expose Lys63 residue within Keap1 for ubiquitination. In addition, Hong et al. (2005) showed that, thiol-reactive electrophile N-iodoacetyl-N-biotinylhexylenediamine (IAB)-induced ubiquitination modification of keap1 at Lys48, Lys298, Cys241, Cys257, and Cys288 can trigger a switching of Cul3-dependent ubiquitination from Nrf2 to Keap1, leading to Nrf2 activation (Figure 1). These ubiquitination sites play a vital role in regulating the interaction between Keap1 and Nrf2. Ubiquitination promotes the binding dissociation between Keap1 protein and Nrf2 protein, allowing Nrf2 protein to escape the inhibition of Keap1 protein, which can enter the nucleus and activate the transcription of antioxidant response genes.

Moreover, the ubiquitination is also able to regulate the stability of the Keap1 protein. Ubiquitination drives the degradation of Keap1 protein, thereby reducing its inhibitory effect on Nrf2 protein. For example, the ubiquitination-dependent degradation of Keap1 causing the loss of Keap1 function further leads to the Nrf2-dependent transcriptional activation of anti-neuroinflammatory genes in microglia, whereas inhibition of ubiquitination-modifying enzymes leads to increased stability of Keap1 protein, enhancing its inhibitory effect on Nrf2 protein (Zhang et al., 2022).

Therefore, studying the mechanism of ubiquitination of Keap1 protein is essential for the thorough comprehension of the molecular mechanism of oxidative stress.

### Glutathionylation

Glutathione is a tripeptide consisting of glutamate, cysteine, and glycine, whose biological function is to maintain the redox state of protein sulfhydryl groups by forming protein-glutathione mixed disulfide (Dalle-Donne et al., 2008). Dalle-Donne et al. (2008) showed that glutathionylation is a specific PTMs of protein cysteine residues, in which glutathione reversibly binds to protein thiols (PSH), producing glutathianylated protein (PSSG).

Carvalho et al. (2016) found that in wild-type mouse model treated with MPTP (1-methyl-4-phenyl-1,2,3,6-tetrahydropyridine), the glutathionylation of Keap1 disrupts the Nrf2-Keap1 complex to enable Nrf2 activation, which increases the expression of HO-1 and glutathione S-transferase pi (GSTP, S-transferase pi) and enhances the antioxidant protective mechanism of the brain to improve Parkinson’s disease (PD). The results of further research by Li et al. (2021) showed that (E)-2-(4-(4 (7 (diethylamino)-2-oxo-2H-chromene-3-carbonyl)-piperazin-1-yl)-styryl)-1,3,3-trimethyl-3H-indol-1-ium iodide (CPC) can regulate Nrf2 nuclear translocation by inhibiting the glutathionization of Cys434 residue of Keap1, and then further regulate autophagy, apoptosis and Nrf2 activity. Therefore, CPC can inhibit Keap1 glutathionylation and promote the interaction between Keap1 and Nrf2. Holland et al. (2008) found the most sensitive cysteine to glutathionylation in the N-terminal, dimerization, central linker, Kelch repeat and C-terminal domain of Keap1, including Cys77, Cys297, Cys319, Cys368, and Cys434. The most readily formed cysteine disulfides are Cys23-Cys38 and Cys257-Cys297 (Figure 1).

The regulation of cellular redox status affects redox-sensitive transcription factors and other stress-related regulatory proteins, and experimental results from Gambhir et al. (2014) showed that 1,4-naphthoquinone (NQ) would induce glutathionylation of Keap1 protein and decrease IKKβ level in lymphocytes when it decreased the ratio of GSH/GSSG (glutathione/oxidized glutathione) to regulate the redox reaction of cells. Further molecular docking studies revealed that NQ could disrupt Keap1/Nrf2 interaction by directly blocking the binding of Nrf2 segments in Keap1 protein and subsequently regulate inflammation and immune responses. Depletion of intracellular GSH by small molecule chemicals that specifically targets GSH is a novel strategy for cancer treatment. Wang et al., 2018 synthesized and demonstrated that a new compound 2-(7-(diethylamino)-2-oxo-2H-chromen-3-yl) cyclohexan-2,5-diamino-1,4-dione (PBQC) can specifically target and deplete GSH in cells, thereby causing glutathionylation conversion of Keap1 protein and promoting Nrf2 nuclear translocation and pro-apoptotic gene expression.

### Alkylation

Protein alkylation refers to the process of adding alkyl groups to amino acid residues in protein molecules, which is a general modification reaction that contributes to the antioxidant mechanism and can affect the protein structure and function (Jourdan et al., 2019). The intervening region (IVR) domain of Keap1 is rich in cysteine residues, once perceived oxidative stress or electrophiles stimulation, one or more of the cysteine thiols will be directly oxidized or alkylated, which in turn leads to reduced ubiquitination and degradation of Nrf2. Newly synthesized Nrf2 continuously accumulates, migrates to the nucleus and activates the downstream ARE, which can upregulates the expression of cytoprotective genes and plays a role in the prevention of degenerative diseases such as cancer (Finkel and Holbrook, 2000; Luo et al., 2007; Mills et al., 2018; Baird and Yamamoto, 2020).

Keap1 protein is rich in 27 cysteines, while Dinkova-Kostova et al. (2002) use mass spectrometry (MS) to map the cysteine modified by dexamethasone 21-mesylate, they found that cysteine residues 257, 273, 288, and 297 located in Keap1 IVR are the sites most susceptible to be alkylated by electrophilic compounds. However, different electrophilic reagents have different alkylation sites for Keap1 cysteine residues. For example, Luo et al. (2007) used a cylindrical ion trap mass spectrometer to perform LC-MS/MS analysis to determine the relative reactivity of three natural electrophilic compounds, xanthohumol, isoliquiritigenin, and 10-shogaol, to specific cysteine residues in human Keap1. It was found that xanthohumol alkylated the Cys151, Cys319, and Cys613 of human Keap1 most easily, isoliquiritigenin alkylated the Cys151 and Cys226 most easily, and 10-shogaol alkylated the Cys151, Cys257, and Cys368. Although all alkylations were specific to cysteines, the alkylation sites of Keap1 to the three electrophiles varied, and it is noteworthy that Cys151 was always detected as the most reactive cysteine.

In a separate study, Mills et al. (2018) conducted an experiment where they overexpressed Keap1 tagged with Myc-DDK in human HEK293T cells and subjected the cells to treatment with 4-octyl itaconate (OI). The findings revealed that OI induced alkylation at the Cys151, Cys257, Cys273, and Cys288 sites of Keap1, leading to an increase in Nrf2 expression and subsequent upregulation of downstream genes associated with antioxidant and anti-inflammatory properties. Consequently, these molecular changes resulted in the manifestation of anti-inflammatory effects. Subsequently, Song et al. (2020) confirmed that itaconate facilitated Nrf2-mediated inhibition of proinflammatory molecules IL-6 and IL-1β through Keap1 alkylation, thereby impeding the development of abdominal aortic aneurysm (AAA) and exerting a protective role against aortic aneurysm. Other electrophiles, such as CDDO (a representative Michael receptor) and DMF (dimethylfumarate), have been reported to modify Keap1 with alkylation (Dunlap et al., 2012; Ahuja et al., 2016). However, the alkylation of Cys14, Cys257, and Cys319 residues on Keap1 induced by CDDO-EA can be inhibited by JFD (a novel biflavonoid isolated from Honeysuckle), leading to ROS accumulation and thereby enhancing *mycobacterium tuberculosis* elimination (Wan et al., 2023).

In addition, the study by Dunlap et al. (2012) revealed that the extent of modification of cysteine residues in different domains of Keap1 varies, even when exposed to the same electrophile. The Keap1 protein was administered with the quinone methide (QM) generated by NO-donating hybrid aspirin prodrug isomer NCX-4040 (pNO-ASA) in the presence of porcine liver esterase (PLE). Subsequently, it was found that the five cysteine residues Cys196, Cys226, Cys249, Cys273, and Cys319 of the central linker domain of Keap1 exhibited QM modification ranging from 8% to 28%, while the N-terminal region of Keap1 observed 19% alkylation only at Cys23, which is the only QM modification outside the central linker domain. Interestingly, neither the BTB nor the Kelch repeat domains show QM modification of cysteine residue.

In summary, alkylation is an important mode of modification of Keap1 by the electrophiles, and the five cysteine residues Cys151, Cys257, Cys273, Cys288, and Cys297 located in the central joint domain of Keap1 are the sites most susceptible to modification by alkylation (Figure 1).

### Glycosylation

Protein glycosylation is one of the most common and significant way of PTMs found in mammals at present, mainly including N-glycosylation, O-glycosylation, glycophospholipid (GPI)-anchored glycosylation and C-glycosylation (Mehboob and Lang, 2021). This type of modification involves the linking of glycans to protein molecules and is required for the proper folding, maintenance of stability and cell adhesion of protein molecules. Among the four types, O-glycosylation or O-linked β-N-acetylglucosaminylation (O-GlcNAcylation) is an essential and nutrient-sensitive PTM that reversibly covalently links β-N-acetylgluglusamine (GlcNAc) to serine and threonine residues of thousands of nuclear proteins, cytoplasmic and mitochondrial proteins (Chen et al., 2017; Dikix, 2017; Xu et al., 2020). The modification process is performed by adding O-linked GlcNAc to the substrate of the uridine diphosphate-GlcNAc donor via a single O-linked β-N-acetylglucosamine transferase (OGT), an intracellular enzyme responsible for glycosylation, and the deglycosylation is removed by a single glycoside hydrolase O-GlcNAcase (OGA) (Chen et al., 2018; Xu et al., 2020).

Previous study has demonstrated that the glycosylation of Keap1 depends on the action of OGT and the acylation of Ser104-specific site is required for the optimal activity of Keap1 in a steady-state environment (Chen et al., 2017). Furthermore, Keap1 is a direct substrate for OGT, and OGT inhibits Nrf2 through glycosylation of Keap1 (Chen et al., 2017; Xu et al., 2020). Xu et al. (2020) determined that overexpression of OGT would increase Keap1 glycosylation, thereby promoting Nrf2 ubiquitination degradation and inhibiting autophagy of vascular smooth muscle cells, and ultimately accelerating hyperphosphate-induced vascular calcification in chronic kidney disease. But this effect was inhibited after mutation of the Keap1 S104 glycosylation site. Proteomic analysis of purified Keap1 based on mass spectrometry by Chen et al. (2017), Chen et al. (2018) identified 11 alternative O-GlcNAcylation sites and found that Keap1 physically interacts with OGT for O-GlcNAcylation in 11 alternative sites. These 11 candidate sites are located in different domains of Keap1. Ser104 and three other putative sites, Ser102, Ser103, Ser166, are located within the α-helices of the BTB domain, which is required for Keap1 homodimerization and interaction with CUL3. Interestingly, when testing the effect of OGT inhibition and Ser104 mutation on Keap1 dimerization, it was found that neither inhibition of OGT nor mutation of Ser104 affected Keap1 dimerization. And the Ser104Ala Keap1 mutant retains the ability to form a dimer with wild-type Keap1. These results indicate that Ser104 glycosylation is not required for Keap1 dimerization (Chen et al., 2017). However, the inhibition of OGT or mutant of Keap1 Ser104 reduces the interaction between Keap1 and CUL3, resulting in subsequent loss of Nrf2 ubiquitination. It is concluded that the main biochemical role of glycosylation at Keap1 Ser104 is to promote its efficient interaction with CUL3, which leads to an effective ubiquitination of Nrf2 (Chen et al., 2017; Chen et al., 2018). Six of the seven additional O-GlcNAcylation sites, Thr388, Ser390, Ser391, Thr400, Ser404, and Ser410, are located in the β-strands of the second Kelch motif, while the last candidate, Ser533, is in the fifth Kelch motif (Figure 1). These glycosylation sites may modulate the activity of Keap1 against Nrf2 or other substrates in response to yet untested stimuli or conditional (Chen et al., 2017).

Keap1 O-GlcNAcylation also correlates with glucose availability (Chen et al., 2017; Chen et al., 2018), that is, the glucose availability moiety is sensed by O-GlcNAcylation of Keap1 specific sites. Low glucose content would reduce Keap1 glycosylation, leading to a decrease in effective Keap1-CUL3 interaction, which activating the Nrf2 pathway. While inhibition of OGA blocks this induction and prevents subsequent Nrf2 signaling.

### Phosphorylation

Protein phosphorylation is an important form of PTMs that is achieved by using phosphorylase to add phosphate groups to specific amino acids of protein (Khoury et al., 2011). It mainly occurs in three amino acids, namely, serine, threonine, and tyrosine. Previous studies have shown that Keap1 amino acid residues are covalently modified in cases of oxidative stress to promote nuclear translocation of Nrf2 (de Freitas Silva et al., 2018; Tu et al., 2019; Baird and Yamamoto, 2020; Ulasov et al., 2022). However, it was unclear until 2018 that whether Keap1 protein occurs in response to oxidative stress and the effect of modification on the interaction between Keap1 and Nrf2.

Wei et al. (2019) simulated the phosphorylation modification by mutating the specific amino acids of the Keap1 protein, and then observed and analyzed the functional significance of Keap1 phosphorylation. Initially, the Halo tag and MS analysis were employed to identify the phosphorylation of Ser53 and Ser293 residues of Keap1 following oxidative stress. After that, the two sites serine and glutamate mutation were constructed in order to introduce negative charge to mimic phosphorylation. The effects of these mutations on the Keap1-Nrf2 complex were then further analyzed. Ultimately, the experimental results showed that although artificial mimic phosphorylation of the two sites has different effects on Keap1-Nrf2 binding, conformational changes after Keap1 modification are more relevant to Ser53, while phosphorylation of Keap1 at Ser53 can enhance the antioxidant capacity of cells to cope with oxidative stress.

### S-sulfhydration

S-sulfhydration is the conversion of cysteine sulfhydryl groups (Cys-SH) into hydropersulfide (Cys-SSH) by the oxidation reduction of H_2_S or persulfide. As a novel PTM, S-sulfhydration encompasses a range of physiological and pathophysiological processes, leading to alterations in the original biological functions of protein molecules (Song et al., 2023). H_2_S is the third member of the family of gas signaling molecules after NO and CO, which plays a wide range of biological roles in the organism. It has been proven to have potent antioxidant and anti-inflammatory properties that can regulate a variety of cardiovascular functions (Xie et al., 2016).

Numerous studies have shown that H_2_S can regulate the intracellular oxidative stress through the S-sulfhydration of the Keap1 protein (Yang et al., 2013). For example, Meng et al. (2017) constructed a mouse model of sulfur mustard (SM)-induced lung injury and found that H_2_S increased the mRNA levels of various downstream protein targeted by Nrf2 such as heme oxygenase-1 (HO-1). The specific mechanism is that H_2_S modifies Keap1 through S-sulfhydration, which then induces Nrf2 dissociation from Keap1 and enhances its nuclear translocation (Buckley et al., 2008; Fujii et al., 2010). Tocmo and Parkin (2019) proved that the onion-derived metabolite S-1-propyylcysteine (CySSPe) can stabilize Nrf2 protein, promote nuclear translocation, and induce the expression of antioxidant enzymes NQO1, HO-1 and GCL. Additionally, it was discovered that cells treated with CySSPe could improve the S-sulfhydration level of H_2_S-induced Keap1 to exert anti-oxidative stress and anti-inflammatory effects. Hourihan et al. (2013) uncovered that H_2_S could inhibit the activation of Keap1 by modifying two sites, 226 and 613, at Keap1 with S-sulfhydration. Yang et al. (2013) clarified the vital role of H_2_S in the mechanism of cellular defense against oxidative stress. Specifically, H_2_S can induce conformation change of Keap1 through S-sulfhydration at Cys151, which subsequently triggers the nuclear translocation of Nrf2 and promotes the dissociation of Nrf2 from Keap1, leading to the activation of Nrf2 and the upregulation of antioxidant genes. Furthermore, in high glucose plus ox-LDL-treated mouse macrophages, Xie et al. (2016) observed that H_2_S activates Nrf2 via increasing S-sulfhydration of Keap1 at Cys151 and Cys273, thereby increasing HO-1 expression and inhibiting O_2_^−^ production, and ultimately improving endothelial function as well as diabetes-accelerated atherosclerosis (Figure 1). Liu et al. (2020) demonstrated that H_2_S mitigates Paraquat-induced acute liver injury by augmenting the antioxidant capacity, regulating mitochondrial function, and inhibiting the activation of the NLRP3 inflammasome induced by ROS. The underlying mechanism involves the promotion of nuclear translocation of Nrf2 and the subsequent activation of Nrf2-driven antioxidant enzymes by NaHS through S-sulfhydration of Keap1. Cui et al. (2021) indicated that GYY4137, through S-sulfhydrylation of Keap1, activates the Nrf2/ARE pathway, leading to anti-inflammatory, anti-apoptotic, and antioxidant effects in septic mice, thereby preserving the integrity of the blood-brain barrier and improving the clinical outcome of sepsis-associated encephalopathy.

The above studies could well elucidate the pivotal role and protective mechanism of H_2_S in the anti-oxidative stress response.

### S-nitrosylation

Protein S-nitrosylation refers to the covalent reaction of nitric oxide (NO) partially coupled to specific protein thiol groups to form S-nitrosothiol, which is a typical redox-dependent protein post-translational modification (Kobayashi et al., 2004; Hess et al., 2005; Nakamura et al., 2018). This is a non-enzymatic reversible process that mainly depends on the proximity of proteins to diffusive NO and plays a key regulatory role in NO-related and redox signaling pathways. For example, bisphenol A (BPA) induces nitric oxide synthase (NOS) to increase NO levels so that the resulting NO modifies Keap1 by S-nitrosylation, leading to Nrf2 stabilization and then inducing the production of Nrf2-dependent drug metabolizing enzymes (Nakamura et al., 2018).

Studies have proved that NO can directly modify thiols of Keap1 with S-nitrosylation, thus affecting the activity of the target protein Nrf2 (Fourquet et al., 2010; Um et al., 2011). It thus suggests that this modifying effect of NO can be used to treat oxidative stress-related diseases. For example, genistein, a natural phytoestrogen, enhances the thiol modification of Keap1 by NO through increasing the activation of endothelial NOS (eNOS), ensuing to upregulates Nrf2/HO-1 antioxidant signaling pathway, and finally exerting a protective effect on delayed neuronal cell death and cognitive decline in the hippocampal CA1 region due to cerebral ischemia (Wang et al., 2013). Long-acting (1R)-isoPropyloxygenipin (IPRG001) has a similar effect with genistein (Koriyama et al., 2010). In addition, luteolin also reduced oxidative stress by enhancing eNOS and increasing the S-nitrosylation of Keap1, which finally protected the heart of diabetic rats from ischemia-reperfusion (I/R) injury (Xiao et al., 2019).

### SUMOylation

SUMO proteins are a series of conserved modifiers of small ubiquitin-like eukaryotic proteins, with four isoforms in the human genome, namely, SUMO1, SUMO2, SUMO3, and SUMO4 (Melchior, 2000; Guo et al., 2004). SUMOylation (Zhao, 2018) is a dynamic and reversible post-translational modification process, which regulates the intracellular distribution of substrate proteins, changes protein conformation or stability and regulates other modifications by covalent binding SUMO proteins to lysine residues of substrate protein with the participation of E1 activase, E2 binding enzyme and E3 ligase.

Since its discovery more than 20 years ago, SUMOylation has been widely recognized, but the SUMOylation of Keap1 has not been recognized. It was not until Yang et al. (2023) found that Keap1 could be modified by SUMO1 and showed for the first time that Keap1 lysine residue 39 (K39) is a modification site targeted by SUMO1 (Figure 1). It was determined that Keap1 activity could also be controlled by the modification of the SUMO proteins. In addition, their group used SUMO1 to treat mutant Keap1 of arginine replacing lysine (K39R) in a subcutaneous tumor model of H1299 lung cancer cell line to study the effect of SUMOylation on activity of Keap1, which showed that the replacement did not affect stability, subcellular localization or dimerization of Keap1. However, compared with unmutated Keap1, K39R mutant Keap1 promoted the formation of Cullin3 ubiquitin ligase and increased Nrf2 ubiquitination, which eventually increased the production of ROS and inhibited tumor growth. In summary, modification of Keap1 K39 site by SUMO1 disrupts the assembly of the potent ubiquitin complex while suppresses its control of Nrf2, promoting expression of Nrf2-targeted genes.

### Other PTMs of Keap1

Apart from the major classes of modification modes described above, Keap1 has some less common or perhaps not currently intensively studied PTMs. For example, methylation, succinylation, S-lactoylation and a novel non-enzymatic PTM of methylimidazole cross-linking.

### Methylation

Protein methylation is a reversible enzymatic PTM, which refers to the transfer of methyl groups to certain residues of proteins (Małecki et al., 2022). It usually occurs on lysine or arginine residues, but also on histidine, cysteine and asparagine, involving important biological processes such as transcriptional activity, signal transduction and regulation of gene expression.

For Keap1, methylation mostly happens on its promoter and is often closely associated with cancer, such as colorectal cancer (Hanada et al., 2012), renal cancer (Fabrizio et al., 2017), and triple-negative breast cancer (TNBC) (Barbano et al., 2013). As for PTM, Wang et al. (2023) revealed that expression of protein arginine methyltransferase 5 (PRMT5) was positively correlated with Keap1 in breast cancer tissues, and high PRMT5 protein levels suggest that TNBC is highly resistant to immunotherapy. The team observed potential interaction between Keap1 and PRMT5 and symmetric methylation of Keap1 by coimmunoprecipitation (co-IP) and mass spectrometry, and this methylation was significantly impaired ensuing to PRMT5 knockdown. It is clear that Keap1 can be methylated by PRMT5 and the stability of modified Keap1 is increased. To further explore the modified site, Keap1 Arg596 was mutated to lysine residue (R596K) and PRMT5 was overexpressed in HEK293T cells. As a result, the ubiquitination of unmutated Keap1 was obviously reduced, but the mutation at R596K could strikingly inhibit this phenomenon. This suggests that PRMT5 binding to R596 on Keap1 inhibits ubiquitination and degradation of Keap1, thereby down-regulating Nrf2 and expression of its downstream gene and promoting the innate resistance of cancer tissues to immunotherapy.

### S-lactoylation

Protein S-lactoylation is a new type of PTM, which was first identified as lysine lactoylation of histone proteins (Zhang et al., 2019), and subsequent studies found that it also exists in non-histone proteins (Gaffney et al., 2020; Gao et al., 2020). A recent study showed that glyceraldehyde 3-phosphoghate (Ga3P) can promote S-lactate modification of Keap1, and this non-enzymatic PTM of cysteine is referred to as S-lactoylation (Ko et al., 2023). To identify the modified sites, they respectively reintroduced single amino acid residues Cys151, Cys273, and Cys288 in cysteine-free Keap1, and then treated HEK293T cells with several different concentrations of sAKZ692 (1, 5, 10, and 20 µM), which is a nonreactive small molecule that promotes Ga3P accumulation. It was observed that all three residues were labeled at high concentrations, but only Cys273 could still be labeled at low concentrations.

### Methylimidazole crosslink of cysteine and arginine

This is a stable and mechanistically novel form of protein PTM, which was initially noticed because some experiments showed that Cys151 was required for Nrf2 activation by oxidants. For example, Um et al. (2011) found that NO promotes the formation of disulfide bonds between two Keap1 molecules via Cys151, thereby releasing and activating Nrf2. Furthermore, Fourquet et al. (2010) found that a portion of Keap1 carried a long-range disulfide bond connecting Cys226 to Cys613 in untreated cells. When cells are exposed to H_2_O_2_ or Cys-NO, the disulfide bond is further increased, and Cys151 promotes the formation of the disulfide bond connecting the two Keap1 molecules. This result indicated that the intermolecular disulfide bond bridging the two monomers of Keap1 based on Cys151 may represent a new modification of the cysteine residue. It was not until Bollong et al. (2018), when explaining the direct link between glycolysis and Keap1-Nrf2 signaling pathway, showed that methylglyoxal (MGx) cross-links the proximal Keap1 molecules in a non-enzymatic manner by a methyl imidazole-based linkage between cysteine (Cys151) and arginine (Arg15 or Arg135) residues, and this novel PTM is termed methyl imidazole-based cross-linking between cysteine and arginine (MICA).

### Succinylation

Protein succinylation is a newly discovered PTM of proteins. It is a process mainly mediated by succinyl-CoA that transfers a negatively charged four-carbon succinyl group to the primary amine of lysine residues, and has important functions and regulatory effects. The mitochondrial metabolites fumarate and succinic anhydride (SA) can both modify Keap1 by succinylation, but at different sites.

Heterozygous mutations in the gene encoding fumarate hydroatase (FH) have been reported to be associated with tumor formation, while high levels of fumarate accumulate and succinylation of protein can readily be detect in both FH-deficient cells and hereditary leiomyomatosis and renal cell carcinoma (HLRCC) (Tomlinson et al., 2002; Bardella et al., 2011; Mitsuishi et al., 2012). Fumarate, an oncogenic metabolite in the TCA cycle, has been shown to modify Keap1 with succinylation at Cys151 and Cys288 (Ooi et al., 2011). Another study proposed the possibility of Nrf2 dysregulation as an alternative oncogenic pathway for FH-related diseases (Adam et al., 2011). They experimentally determined that fumarate accumulation due to FH deficiency causes succinylation of Keap1 and that the pathophysiological levels of fumarate associated with cancer are sufficient to make Keap1 succinylation and activate Nrf2 signaling. In addition, succinylation at Keap1 residues Cys38, Cys151, Cys241, Cys288, Cys319, and Cys613 were identified (Figure 1).

Unlike fumarate that modifies cysteine residues, SA modifies the lysine residue of Keap1. Ibrahim et al. (2023) determined that genetic depletion of succinyl-CoA synthetase (SCS) leads to succinylation of Keap1 at Lys131 site and subsequent activation of Nrf2, and then found that the modification actually originated from SA. SA is a highly reactive cyclic byproduct generated by self-hydrolysis succinyl-CoA (Wagner et al., 2017). The experimental results showed that the consumption of SCS would increase the concentration of SA, while the covalent modification of Keap1 by SA disrupted the interaction between Keap1 and CUL3 and activated the ARE gene. This is yet another endogenous reactive metabolite that links metabolism to oxidative stress.

### PTMs of Keap1 and aging

The available body of evidence indicates a decline in Nrf2 levels with advancing age, while also establishing a positive correlation between Nrf2 activity and the lifespan of various species. Consequently, the regulation and subsequent pathways of Nrf2 have garnered significant attention. Aging is closely linked to inflammation, which generates substantial quantities of ROS capable of instigating oxidative harm to DNA, membrane lipids, and proteins, thereby exacerbating the aging process (Sendama, 2020; Shang et al., 2020). Itaconate has been identified as a potent and novel activator of Nrf2, which exerts its anti-inflammatory effects by alkylating specific cysteine residues in Keap1. This alkylation enables Nrf2 to enhance the expression of downstream genes involved in antioxidant and anti-inflammatory processes (Mills et al., 2018). One derivative of itaconate, known as 4-octyl itaconate (OI), has been found to alkylate Keap1 at Cys151, and subsequent activation of Nrf2 may be the primary mechanism underlying OI-induced neuroprotection against H_2_O_2_ (Liu et al., 2018).

Collectively, these findings indicates that Keap1 serves as a crucial component of the Keap1-Nrf2 system, which safeguards cells against oxidative harm by sensing oxidative stress and modulating Nrf2 activity. Investigations into the PTMs of Keap1 play a pivotal role in elucidating the underlying mechanisms of aging and in the development of agents against aging.

## Conclusion

**FIGURE 2:**
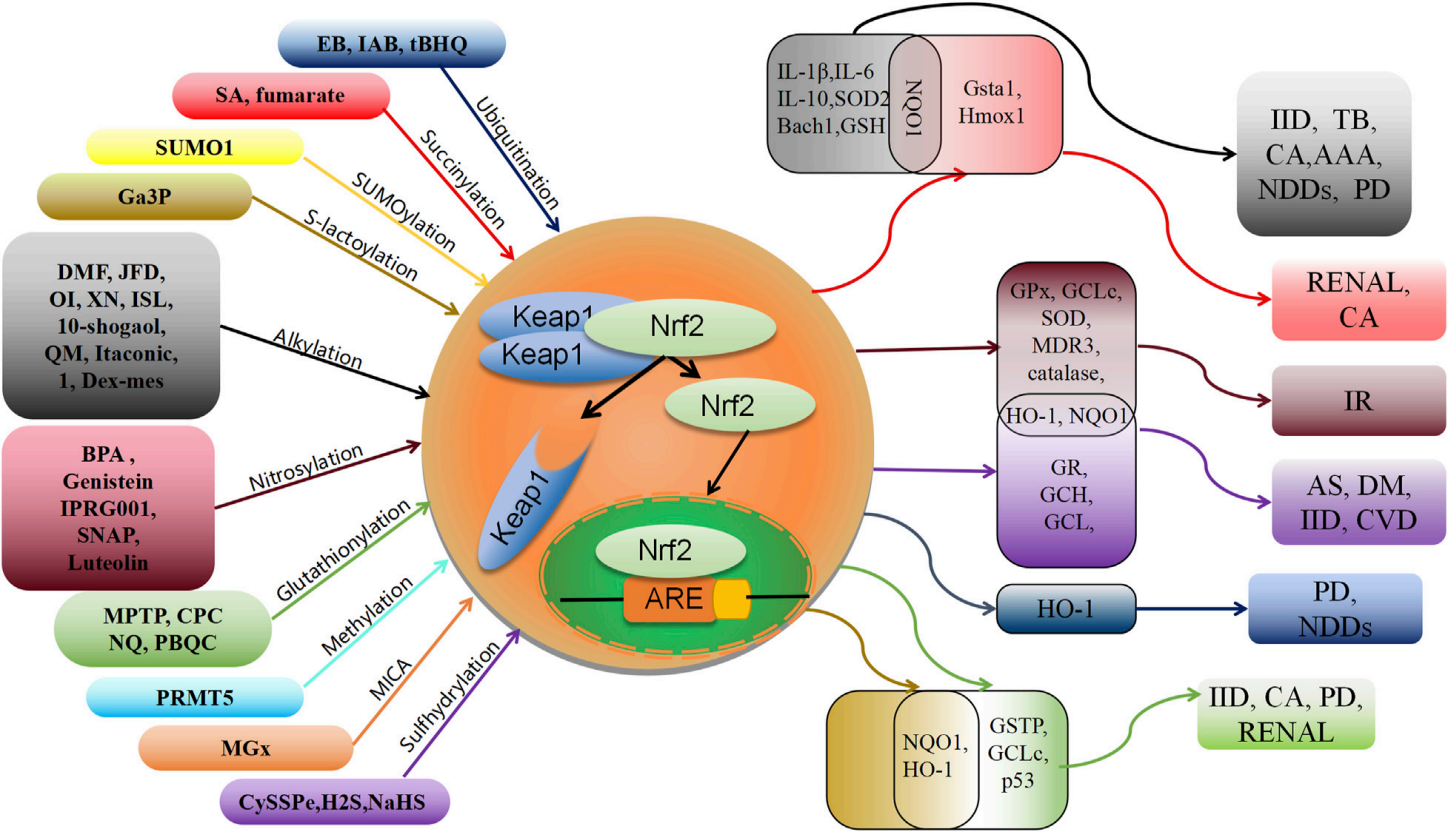
Schematic association of post-translational modifications of Keap1 with associated oxidative stress diseases. XN: xanthohumol; ISL: isoliquiritigenin; NDDs: Neurodegenerative diseases; PD: Parkinson’s disease; IID: Inflammatory and immune diseases; CA: cancer; TB: tuberculosis; AAA: abdominal aortic aneurysm; AS: Atherosclerosis; IR: Ischemia-reperfusion; DM: diabetes; CVD: cardiovascular disease.

This review presents a comprehensive overview of the existing research advancements concerning the post-translational modifications (PTMs) of Keap1. Specifically, it encompasses the recently identified PTMs patterns, the locations of modification sites, and the resultant impact on the Nrf2 pathway (Table 1). These investigations establish a solid theoretical foundation for the therapeutic and interventional approaches targeting Keap1 PTMs in oxidative stress-related ailments. Notably, numerous naturally occurring compounds derived from plants, such as genistein (Tocmo and Parkin, 2019), Japoflavone D (Wan et al., 2023), and luteolin (Nakamura et al., 2018), have been experimentally validated to exhibit favorable effects on diseases through the activation of the Nrf2 pathway in both cellular and animal models. However, it should be noted that these substances do not directly alter or engage with Nrf2. Instead, they indirectly impact Nrf2 by regulating the PTMs of Keap1. Consequently, they hold potential for the treatment of oxidative stress-related ailments like diabetic heart (I/R) injury (Nakamura et al., 2018), tuberculosis (Wan et al., 2023), and cerebral ischemia cognitive decline (Tocmo and Parkin, 2019) (Figure 2). This suggests that Keap1 could be a promising therapeutic target.

**TABLE 1:** Keap1 post-translational modification.

Modifications	Materials	Sites	Function	References
Ubiquitination	—	I125A, E126A, G127A, Y162A, Q163A, I164A	ARE, Nrf2↑	Zhang et al. (2004)
	EB	—	Keap1↓	Zhang et al. (2022)
	tBHQ	C151, K63	Keap1↓	Zhang et al. (2005)
	IAB	K48, K298, C241 C257, C288	Keap1↓Nrf2, HO-1↑	Hong et al. (2005)
Glutathionylation	MPTP	C434	HO-1, GSTP↑	Carvalho et al. (2016)
	CPC	C434	Nrf2, Keap1↑	Li et al. (2021)
	—	C77, C29, C319, C368, C434	Proteases↓	Holland et al. (2008)
	NQ	—	Nrf2, GCLc, HO-1↑	Gambhir et al. (2014)
	PBQC	—	HO-1, GCLc, NQO1, p53↑ ROS↓	Wang et al. (2018)
Alkylation	DMF	C151	Bach1↑	Ahuja et al. (2016)
	JFD	C14, C257, C319,	Nrf2, SOD2↓, ROS↑	Wan et al. (2023)
	OI	C151, C257, C288, C273, C297	Nrf2↑IL-1β, IL-10↓	Mills et al. (2018)
	xanthohumol isoliquiritigenin 10-shogaol	C151, C319, C613 C151, C226, C151, C257, C368	ARE↑	Luo et al. (2007)
	QM	C23, C196, C226, C249, C273, C319	ARE, NQO1, GSH↑	Dunlap et al. (2012)
	1, Dex-mes	C257, C273, C288, C297	ARE↑	Dinkova-Kostova et al. (2002)
	Itaconate	C151	IL-6, IL-1β↑	Song et al. (2020)
Glycosylation	OGT	S104	Nrf2↓	Xu et al. (2020)
	OGT	S102, S103, S104, S166, S390, S391, S404, S410, S533, T388, T400	Nrf2↓	Chen et al. (2017)
Phosphorylation	—	S53	—	Wei et al. (2019)
S-sulfhydrytion	NaHS	C151	Nrf2, GCL, GR, GCH↑	Yang et al. (2013)
	H2S	C151, C273	HO-1↑, ROS↓	Xie et al. (2016)
	NaHS	C226, C613	ROS↓	Hourihan et al. (2013)
	CySSPe	—	NQO1, HO-1, GCL↑	Tocmo and Parkin (2019)
	H2S	C151	Nrf2↑ROS↓	Meng et al. (2017)
	NaHS	—	NQO1, HO-1, SOD↑	Liu et al. (2020)
	GYY4137	—	Nrf2, ARE, NQO1, HO-1↑	Cui et al. (2021)
SUMOylation	SUMO1	K39	ROS↑	Yang et al. (2023)
S-nitrosylation	BPA	—	Nrf2, HO-1, MDR3↑	Nakamura et al. (2018)
	Genistein	—	Nrf2/HO-1↑	Wang et al. (2013)
	IPRG001	—	HO-1, NQO-1,GCLc↑	Koriyama et al. (2010)
	Luteolin	—	ROS↓ HO-1, SOD, GPx↑	Xiao et al. (2019)
	SNAP	—	HO-1↑	Um et al. (2011)
Methylation	PRMT5	R596	Nrf2/HO-1↓	Wang et al. (2023)
S-lactoylation	Ga3P	C273	NQO1, HO-1 ↑	Gaffney et al. (2020)
Succinylation	SA	K131	ARE↑	Adam et al. (2011)
	fumarate	C38, C151, C241, C288, C319, C613	Nrf2, Gsta1, Hmox1, Nqo1↑	Ooi et al. (2011)
	fumarate	C151, C288	ARE↑	Bardella et al. (2011)
MICA	MGx	C151-R15 or C151-R135	ARE↑	Bollong et al. (2018)

EB: eupalinolide B; tBHQ: tert-butylhydroquinone; IAB: N-iodoacetyl-N-biotinylhexylenedi-amine; MPTP: 1-methyl-4-phenyl-1,2,3,6-tetrahydropyridine; CPC: (E)-2-(4-(4-(7-(diethylamino)-2-oxo-2H-chromene-3-carbonyl)-piperazin-1-yl)-styryl)-1,3,3-trimethyl-3H-indol-1-ium iodide; NQ: 1,4-naphthoquinone; PBQC: 2-(7-(diethylamino)-2-oxo-2H-chromen-3-yl)cyclohexa-2,5-die-ne-1,4-dione; DMF: dimethylfumarate; JFD: Japoflavone D; QM: quinone methide; OGT: O-linked N-acetylglucosamine transferase; CySSPe: S-1-propenylmercaptocysteine; BPA: Bisphe-nol A; IPRG001: Long-acting (1R)-isoPropyloxygenipin; SNAP: S-nitroso-N-acetylpenicillamine; PRMT5: otein arginine methyltransferase 5; MGx: methylglyoxal.

Nonetheless, several concerns must be taken into account when developing drugs based on the PTMs of Keap1 to facilitate future clinical applications. Firstly, the activation of downstream antioxidant genes in the Keap1-Nrf2 pathway is primarily mediated by Nrf2, while the regulation of Nrf2 itself is not solely dependent on Keap1 (Liu et al., 2021b). Additionally, excessive activation of Nrf2 can lead to physiological abnormalities, underscoring the importance of controlling drug release to minimize adverse reactions. Secondly, recent discoveries have unveiled novel types of PTMs such as S-lactoylation (Ko et al., 2023) and MICA (Bollong et al., 2018), with the modifiers identified in relevant studies being derivatives involved in glucose metabolism. Other metabolic derivatives, such as fumarate (Adam et al., 2011; Ooi et al., 2011), have been identified as potential mechanisms underlying the development of HLRCC through the modification of Keap1 via succinylation. These findings suggest that disrupted glucose metabolism may contribute to cancer development by interfering with the antioxidant stress pathway, making PTMs of Keap1 a promising area of investigation. Additionally, the gasotransmitters NO and H_2_S have been found to modify Keap1 through nitrosylation and sulfhydration, respectively. Recent advancements in the study of NO and H_2_S have led to the development of various donors and targeted prodrugs, such as NO donor S-nitroso-N-acetylenemethamine (SNAP) (Um et al., 2011), H_2_S donor S-1-propyl cysteine (CySSPe) (Tocmo and Parkin, 2019), and mitochondrial-targeted H_2_S prodrugs AP39 and RT01 (Magierowska et al., 2022), further expanding our understanding of their biological effects. The aforementioned studies offer valuable insights for the development of pharmaceuticals that stimulate the Nrf2 pathway.

Finally, a comprehensive examination of modification sites is imperative for the formulation of targeted medications. While numerous enzymes (Chen et al., 2017; Wang et al., 2023) and TCA cycle derivatives (Adam et al., 2011; Song et al., 2020) can induce various PTMs on Keap1, the majority of these modifications predominantly occur at Cys151. Recent investigations have identified Cys151 as a crucial determinant of oxidative stress and the most frequently modified site (Zhang and Hannink, 2003). Drug development targeting this locus has the potential to greatly enhance the treatment of patients with multiple diseases. However, further research is required to determine whether there will be any side effects in patients with a single disease. In summary, our objective is not only to pursue multiple treatments with a single drug, but also to prioritize specific drugs for specific diseases.
