# Supplementary material for: Post-translational modifications of Keap1: the state of the art
Source: Front Cell Dev Biol. 2024 Jan 8;11:1332049. doi: 10.3389/fcell.2023.1332049 (PMC10801156; doi:10.3389/fcell.2023.1332049)
Supplement: Supplementary file 1 [file Table1.docx]

| **Modifications** | **Materials** | **Sites** | **Function** | **References** |
| --- | --- | --- | --- | --- |
| Ubiquitination | — | I125A, E126A, G127A,  Y162A, Q163A, I164A | ARE, Nrf2↑ | Zhang et al. (2004) |
|  | EB | — | Keap1↓ | Zhang et al. (2022) |
|  | tBHQ | C151, K63 | Keap1↓ | Zhang et al. (2005) |
|  | IAB | K48, K298, C241  C257, C288 | Keap1↓Nrf2, HO-1↑ | Hong et al. (2005) |
| Glutathionylation | MPTP | C434 | HO-1, GSTP↑ | Carvalho et al. (2016) |
|  | CPC | C434 | Nrf2, Keap1↑ | Li et al. (2021) |
|  | — | C77, C29,  C319, C368, C434 | Proteases↓ | Holland et al. (2008) |
|  | NQ | — | Nrf2, GCLc, HO-1↑ | Gambhir et al. (2014) |
|  | PBQC | — | HO-1, GCLc, NQO1, p53↑ ROS↓ | Wang et al. (2018) |
| Alkylation | DMF | C151 | Bach1↑ | Ahuja et al. (2016) |
|  | JFD | C14, C257, C319, | Nrf2, SOD2↓, ROS↑ | Wan et al. (2023) |
|  | OI | C151, C257, C288, C273, C297 | Nrf2↑IL-1β, IL-10↓ | Mills et al. (2018) |
|  | xanthohumol  isoliquiritigenin  10-shogaol | C151, C319, C613  C151, C226, C151, C257,C368 | ARE↑ | Luo et al. (2007) |
|  | QM | C23, C196, C226, C249, C273, C319 | ARE, NQO1, GSH↑ | Dunlap et al. (2012) |
|  | 1, Dex-mes | C257, C273, C288, C297 | ARE↑ | Dinkova-Kostova et al. (2002) |
|  | Itaconate | C151 | IL-6, IL-1β↑ | Song et al. (2020) |
| Glycosylation | OGT | S104 | Nrf2↓ | Xu et al. (2020) |
|  | OGT | S102, S103, S104, S166, S390, S391, S404, S410, S533, T388, T400 | Nrf2↓ | Chen et al. (2017) |
| Phosphorylation | — | S53 | — | Wei et al. (2019) |
| S-sulfhydrytion | NaHS | C151 | Nrf2, GCL, GR, GCH↑ | Yang et al. (2013) |
|  | H_2_S | C151, C273 | HO-1↑, ROS↓ | Xie et al. (2016) |
|  | NaHS | C226, C613 | ROS↓ | Hourihan et al. (2013) |
|  | CySSPe | — | NQO1, HO-1, GCL↑ | Tocmo et al. (2019) |
|  | H_2_S | C151 | Nrf2↑ROS↓ | Meng et al. (2017) |
|  | NaHS | — | NQO1, HO-1, SOD↑ | Liu et al. (2020) |
|  | GYY4137 | — | Nrf2, ARE, NQO1, HO-1↑ | Cui et al. (2021) |
| SUMOylation | SUMO1 | K39 | ROS↑ | Yang et al. (2023) |
| S-nitrosylation | BPA | — | Nrf2, HO-1, MDR3↑ | Nakamura et al. (2018) |
|  | Genistein | — | Nrf2/HO-1↑ | Wang et al. (2013) |
|  | IPRG001 | — | HO-1, NQO-1,GCLc↑ | Koriyama et al. (2010) |
|  | Luteolin | — | ROS↓ HO-1, SOD, GPx↑ | Xiao et al. (2019) |
|  | SNAP | — | HO-1↑ | Um et al. (2011) |
| Methylation | PRMT5 | R596 | Nrf2/HO-1↓ | Wang et al. (2023) |
| S-lactoylation | Ga3P | C273 | NQO1, HO-1 ↑ | Gaffney et al. (2020) |
| Succinylation | SA | K131 | ARE↑ | am et al. (2011) |
|  | fumarate | C38, C151, C241,  C288, C319, C613 | Nrf2, Gsta1, Hmox1, Nqo1↑ | Ooi et al. (2011) |
|  | fumarate | C151, C288 | ARE↑ | Bardella et al. (2011) |
| MICA | MGx | C151-R15 or C151-R135 | ARE↑ | Bollong et al. (2018) |

EB: eupalinolide B; tBHQ: tert-butylhydroquinone; IAB: N-iodoacetyl-N-biotinylhexylenedi-amine; MPTP: 1-methyl-4-phenyl-1,2,3,6-tetrahydropyridine; CPC: (E)-2-(4-(4-(7-(diethylamino)-2-oxo-2H-chromene-3-carbonyl)-piperazin-1-yl)-styryl)-1,3,3-trimethyl-3H-indol-1-ium iodide; NQ: 1,4-naphthoquinone; PBQC: 2-(7-(diethylamino)-2-oxo-2H-chromen-3-yl)cyclohexa-2,5-die-ne-1,4-dione; DMF: dimethylfumarate; JFD: Japoflavone D; QM: quinone methide; OGT: O-linked N-acetylglucosamine transferase; CySSPe: S-1-propenylmercaptocysteine; BPA: Bisphe-nol A; IPRG001: Long-acting (1R)-isoPropyloxygenipin; SNAP: S-nitroso-N-acetylpenicillamine; PRMT5: otein arginine methyltransferase 5; MGx: methylglyoxal.
